# Research on real-world emission characteristics based on the Symmetry Solid SCR system

**DOI:** 10.1371/journal.pone.0320323

**Published:** 2025-04-29

**Authors:** Yingshuai Liu, Jianwei Tan, Chenxing Liu, Yunli He

**Affiliations:** 1 Shandong Huayu University of Technology, Dezhou, China; 2 National Lab of Auto Performance and Emission Test, School of Mechanical and Vehicular Engineering, Beijing Institute of Technology, Beijing, China; Marwadi University, INDIA

## Abstract

In the actual use process, especially under the low exhaust temperature in urban conditions, the actual NOx emission of diesel engines using the Urea SCR technology is higher than expected, which seriously affects the application of Urea SCR technology in urban diesel vehicles. To solve the defects of low-temperature freezing, urea crystallization in the exhaust pipeline at low exhaust temperature, and insufficient activity of the Urea SCR system at low exhaust temperature in the actual application process of the Urea SCR technology, this paper uses the Portable Emission Measurement System (PEMS) vehicle test system to run the real-world. A SAIC Hongyan Dump truck that meets the China VI emission standard is tested and studied, and the real-world emission characteristics of the Solid SCR system and the Urea SCR system are compared and studied. Among other things, solid-state SCR systems use solid ammonium as a precursor to ammonia, a change that reduces clogging problems due to icing and hydrolysis evaporation crystals at low temperatures compared to conventional aqueous urea solutions. This results in more reliable system operation in cold environments. Solid-state SCR technology offers a more efficient and reliable solution for diesel exhaust treatment through its unique ammonia supply method, system optimization and environmental adaptability, helping to reduce environmental pollution from diesel vehicles. The test results show that: Under real-world operation conditions, the NOx conversion efficiency of the Urea SCR system and the Solid SCR system with the same injection strategy is increased by 0.5% and the NOx emission is reduced by 8.9% when the window average temperature is 228.8°C and 231.3°C. For the PN emission, no matter whether the Urea SCR system or the Solid SCR system is selected, the results have little influence. Based on the Solid SCR system, CO, HC, NO, NO_X,_ and other pollutant emission factors gradually decrease and tend to be relatively stable with the increase in vehicle speed. The Solid SCR technology is a promising NOx emission control technology for diesel engines.

## Introduction

Automobiles have promoted the rapid development of society, improved people’s quality of life, and contributed to the national economy, but their rapid development has also brought negative environmental impacts. Pollutants such as CO, HC, NOx and PM emitted by vehicles have an increasing impact on humans and the environment [1–3]. Among them, NOx (nitrogen oxide) emissions in the urban environment mainly come from diesel driven heavy trucks, buses and construction machinery. These vehicles and equipment play an important role in urban transportation, public transportation, and construction operations, but at the same time introduce significant NOx emission problems. NOx emission has a serious impact on the health of urban residents and the environment. It can cause respiratory and cardiovascular diseases and increase the health risk of residents. At the same time, NOx is also an important factor in the formation of environmental problems such as acid rain and photochemical smog. Therefore, the problem of NOx emissions from diesel in the urban environment needs to be solved urgently to reduce its negative impact on residents’ health and the environment [4,5].

Due to the combustion mode of diesel engines and the “trade-off” relationship between NOx and PM generated in the cylinder, diesel engine emissions are characterized by low CO and HC emissions and high NOx and PM emissions [6–8]. It can be seen that the NOx/PM emission of diesel vehicles is the main source of NOx/PM emission of vehicles, so the control of NOx and PM emission of diesel vehicles has become the focus of automobile pollutant emission control.

Urea SCR is a common vehicle exhaust treatment. Above 180°C, urea hydrolyzes to NH_3_, reacting with NOx to reduce emissions[9–11]. However, the application of the Urea SCR technology in the after-treatment of motor vehicle exhaust gas has the following difficulties: In actual use, especially in light and heavy diesel vehicles running in cities with low exhaust temperature for a long time, the Urea SCR system cannot play a role in most of the time because urea can be hydrolyzed to NH_3_ only when it reaches 180°C, and the actual NOx emission is far higher than the emission limit, leading to the frequent torsional limit alarm of vehicles [12–15]. In contrast, the Solid SCR technology is a new technology to reduce NOx emissions that have emerged in recent years. It can be applied to low-temperature conditions where the Urea SCR system cannot work and can improve the NOx emission performance of diesel engines at low temperatures [16–18]. Li Jianli, Ge Yunshan et al studied the NOx emission characteristics of the Urea SCR system and the Solid SCR system on the diesel engine bench, and the research results showed that Solid SCR system would not produce urea crystallization phenomenon, and the application of the Solid SCR technology at low and medium exhaust temperature could improve the NOx emission of diesel engine [19]. Liu Ming, Ma Yi et al studied the real-world emissions of diesel vehicles equipped with the Solid SCR based on a feature map, and the results showed that the application of the Solid SCR technology greatly reduced NOx emissions [20]. Liu Ming, He Chao, et al analyzed the influence law of the Solid SCR on diesel vehicle NOx emission by using the emission data obtained from automobile exhaust real-time monitoring platform, and the research results showed that Solid SCR can greatly improve the NOx emission of diesel vehicle when the exhaust temperature is low [21]. Zhang Kejin, Cui Long et al also carried out relevant studies on the development of solid ammonia storage materials suitable for SCR [22].

The Solid SCR technology stores NH_3_ in the form of a solid ammonium salt and keep it in a closed container. When heated to a certain temperature, solid ammonium salt will release the stored NH_3_ and quantitatively inject NH_3_ into the exhaust pipe according to the requirements of engine working conditions. Under the action of SCR catalyst, NH_3_ reacts with NOx in exhaust [23–26]. Typical ammonium-storage materials, such as carbamate and ammonia carbonate, decompose when heated in a closed container until the vapor pressure of NH_3_ and CO_2_ reaches equilibrium, at which point no further decomposition occurs unless temperature increases or the gas escapes [27–29]. After cooling, the process is reversed, reforming the original ammonium salt [30–33]. The chemical formula of NH_3_ generated by ammonium salt reaction is shown in Equations (1–4):


$$NH_{4}COONH_{2}↔2NH_{3}+CO_{2}$$ (1)

$${\left(NH_{4}\right)}_{2}CO_{3}↔2NH_{3}+CO_{2}+H_{2}O$$ (2)

$${\left(NH_{4}\right)}_{2}CO_{3}↔NH_{3}+\left(NH_{4}\right)HCO_{3}$$ (3)

$$\left(NH_{4}\right)HCO_{3}↔NH_{3}+CO_{2}+H_{2}O$$ (4)

To sum up, the Solid SCR technology can solve the defects of current Urea SCR technology such as insufficient low-temperature activity, crystallization of exhaust pipes, and low-temperature icing. It is a very promising diesel engine NOx emission control technology [34–36].

However, current experimental research mostly relies on test benches, failing to accurately simulate real-world NO_X_ emissions. Evaluating Solid SCR technology’s effect on heavy-duty diesel engines’ NOx control in real conditions is needed, this test was performed in a unit equipped with Diesel Oxidation Catalyst (DOC)+ Diesel Particulate Filter (PDF) + Selective Catalytic Reduction (SCR) + Ammonia Slip Catalyst (ASC) used the PEMS on the China VI SAIC Hongyan Dump truck to compare and study the pollutant emission characteristics of urban heavy-duty diesel vehicles using The Urea SCR technology and the Solid SCR technology under real-world operating conditions.

## Materials and methods

### Test vehicle

In the on-board test, the model selection of the test vehicle fully considered the representativeness of the vehicle in the local municipal traffic, and a SAIC Hongyan Dump truck that reached the China VI emission standard was selected as the vehicle test object. The main parameters of the test vehicle are shown in Table 1. The after-treatment technology system of the test vehicle is DOC+DPF+SCR+ASC. During the test, the system works normally, and no alarm or fault affects the normal operation of the system.

**Table 1:** Main parameters of the test vehicle.

Project	Parameter
Manufacturer	Shanghai Iveco Hongyan Commercial Vehicle Co., Ltd.
Production time	2021.5
Engine type	Diesel engine, in-line 6-cylinder, supercharged and intercooler, high-pressure common rail
Engine displacement (L)	8.7
Engine rated power/ speed kw/ (r.min^-1^)	257/2100
Maximum net engine power/ speed kw/ (r.min^-1^)	255/2100
Maximum total mass (kg)	12500
Emission Standards	China VI
Aftertreatment system	DOC+DPF+SCR+ASC

During the on-board test, to simulate the vehicle load, the test vehicle is equipped with a sandbag of about 8000 kg as the load. The total weight of the whole set of test equipment (PEMS/generator/battery), load, and on-board test testers is about 2000 kg. The car air conditioner is turned on throughout the experiment to simulate about 60% of the effective load of the test vehicle.

### Test route

To measure the pollutant emission of the vehicle under the actual operating conditions of the urban road, the vehicle emission test route selects the normal driving road conditions of the vehicle, and the test route selects the urban road of Zhangjiakou, and the driving route includes the urban main roads (Hanghai East Road, Zhongzhou Avenue, South The Third Ring Road), urban secondary roads (Shangdu Road) and expressways (Beijing-Hong Kong-Macao Expressway) form a loop with a total length of 26.8km. This route is designed to simulate normal driving conditions on urban roads, where the proportion of urban main roads, secondary roads and highways is 50%, 30% and 20% respectively. The high proportion of urban main and secondary roads reflects the characteristics of frequent starts and stops and low speed driving in urban traffic.

During the on-board test, according to factors such as the maximum speed of the test vehicle, road traffic intensity, and road intersection density distribution, the test line is divided into three parts: urban road, suburban road, and highway. The composition of each part and the ratio of road length are shown in Table 2.

**Table 2:** Composition of the test route.

Road type	Maximum speed (km.h^-1^)	Road length (km)	Proportion
Urban road	40	13.3	49.6%
Suburban road	60	10.2	38.1%
Highway	90	3.3	12.3%

### Vehicle test equipment

The PEMS vehicle test system is mainly composed of a GAS PEMS gaseous pollutant emission analyzer, PN PEMS particulate matter analyzer, EFM (Exhaust Flow Meter), notebook computer, and GPS (Global Positioning System). Among them, the GAS PEMS gaseous pollutant emission analyzer is produced by Austrian AVL Company, which is used to measure the concentration of gaseous pollutants such as CO_2_, CO, NOx, and THC in the exhaust gas of the engine, using the NDIR (Non-Dispersive Infrared, non-spectrographic infrared analyzer) to measure the volume concentration of CO_2_ and CO components, using HFID (Heated Flame Ionization Detector, heating type hydrogen flame ionization analyzer) to measure the THC composition in the exhaust gas. The EFM is used to measure the instantaneous exhaust flow of the engine during the on-board test, and the PN PEMS particle analyzer is used to measure the amount of particulate matter during the on-board test. During the test, GPS is used to record the driving state parameters of the test vehicle, such as vehicle speed, altitude, and vehicle driving position. GAS PEMS also utilizes some external sensors to measure environmental state parameters during the test, such as atmospheric pressure, temperature, and relative humidity.

To prevent distortion of test results due to condensation of pollutant components, GAS PEMS uses a heated sampling line to sample engine exhaust GAS at a temperature of approximately 190 °C. To ensure the normal operation of the on-board emission testing equipment during the test, each GAS PEMS measurement module and the heated sampling pipeline should be preheated for about one hour before use. After the analyzer is preheated, leak detection should be performed on GAS PEMS and sampling pipelines first, and then zero calibration of NDUV, NDIR, FID, and other measurement modules should be performed using pure N_2_ standard gas. NO, NO_2_, THC, CO_2_, CO, and other pollutants were calibrated with standard distance gas to ensure the accuracy of the measurement results. Table 3 lists the measurement range, measurement accuracy, and resolution of each GAS PEMS module.

**Table 3:** Performance parameters of the GAS PEMS.

Component of measurement	Analysis module	Measuring range	Resolution	measurement accuracy	Response time
NO	NDUV	0-2500ppm	1ppm	±15ppm or ±3%	≤2s
NO_2_	NDUV	0-500ppm	1ppm	±10ppm or ±3%	≤2s
CO_2_	NDIR	0-20%	0.01%	±3%	≤3s
CO	NDIR	0-8%	10ppm	±50ppm or ±3%	≤3s
		0-100ppm	0.1ppm	±5ppm or ±2%	
THC	HFID	0-1000ppm	1ppm	±5ppm or ±2%	≤2s
		0-10000ppm	1ppm	±25ppm or ±2%	

During the on-board test, GAS PEMS and EFM were used to measure and record the instantaneous exhaust flow, pollutant emission concentration, and environmental state parameters of the test vehicle, such as atmospheric temperature, atmospheric pressure, and relative humidity, to calculate the instantaneous pollutant emission of the test vehicle. PN PEMS was used to measure the number of particles in the on-board experiment. GPS is used to record the instantaneous speed and longitude and latitude information of the test vehicle. In the on-board test, an SCR catalyst upstream NOx sensor was installed in the upstream position of the urea nozzle of the engine exhaust pipe to compare and study the NOx emission levels of the engine in different test cycles and evaluate the control effect of the Urea SCR system and the Solid SCR system on the NOx emission of the original engine under actual driving conditions. According to EFM instantaneous exhaust flow, the real-time NOx emission of the original machine can be calculated.

### SCR system control mode

DOC+DPF+SCR+ASC is the after-treatment technology system of the heavy diesel bus of the test vehicle, in which the SCR system is equipped with a copper molecular sieve catalyst. To compare the control effect of the Urea SCR system and the Solid SCR system on NOx emission of the engine of the test vehicle under real-world driving conditions, a Solid SCR system was also installed in the after-treatment system of the test vehicle. The two sets of SCR systems can choose to use the Urea SCR system or the Solid SCR system to control the NOx emission of the test vehicle by switching different control modes, to analyze and evaluate the actual control effect of different SCR systems on NOx emission of the engine of the test vehicle.

The basic function of the injection control strategy of the Urea SCR system is to quantitatively inject urea aqueous solution into the exhaust pipe. Under the action of the exhaust temperature, the NOx emission of the engine can be controlled to meet the emission limits of corresponding pollutants, and the requirements of ammonia leakage limits of the regulations can be met at the same time. In the on-board test, the Urea SCR system of the test vehicle applied an open-loop control strategy to control the quantitative injection of urea solution. The injection control strategy calculates the basic urea injection amount according to the NOx emission concentration of the engine, the exhaust gas flow rate, the target conversion efficiency of the catalyst, and the stoichiometric ratio of urea to NOx. A series of correction coefficients were introduced to modify the basic urea injection volume to obtain the revised urea injection volume, such as catalyst temperature correction coefficient, exhaust flow correction coefficient, exhaust molar mass correction coefficient, catalyst deterioration coefficient, and engine deterioration coefficient. Finally, to control the ammonia leakage discharge that may occur in the transient process, the transient correction coefficient is introduced to modify the urea injection amount and obtain the final urea injection demand.

According to the corresponding engine’s original NOx emission MAP, tail gas flow MAP, and SCR catalytic converter conversion efficiency MAP table lookup table, the starting engine NOx emission concentration, tail gas flow, NOx conversion efficiency, and other parameters can be calculated. Considering the influence of engine inlet state parameters and cooling water temperature on engine NOx primary exhaust, the correction coefficient of inlet temperature, inlet pressure, and the cooling water temperature were introduced to correct engine NOx primary exhaust.

In the on-board test, to compare the NOx emission of the Urea SCR system and the Solid SCR system in the real-world operation condition of the test vehicle, the Urea SCR system, and the Solid SCR system should adopt the same injection strategy. To achieve the same injection control strategy, the SCR after-treatment system adopts a Master-Slave Control Mode to achieve the same control strategy between the Urea SCR system and the Solid SCR system.

In the on-board test, when the Master Control Mode is activated, the Urea SCR system works alone, while the Solid SCR system does not. DCU performs urea injection according to the injection strategy of the Urea SCR system. At this time, the on-board test is to test the NOx emission of the test vehicle under the real-world operation condition when the Urea SCR system is applied. When the Slave Control Mode is activated, the Solid SCR system and the Urea SCR system work simultaneously. The control unit ACU of the Solid SCR system reads the urea injection quantity signal of the DCU of the Urea SCR system. According to Equation 5, the amount of reducing agent NH_3_ injection is determined (the ratio of urea completely decomposed into reducing agent NH_3_ is 1:2 under ideal conditions):


$$Q_{NH_{3}}=Q_{Urea}\times \rho_{Urea}\times \omega_{Urea}\times 2M_{NH_{3}}/M_{Urea}$$ (5)

where $Q_{NH_{3}}$ is the NH_3_ injection amount of the Solid SCR system, in mg.s^-1^; $Q_{Urea}$ is the injection volume of urea aqueous solution in the Urea SCR system, in ml.h^-1^; $\rho_{Urea}$ is the density of urea aqueous solution, take 1.1g.ml^-1^; $\omega_{Urea}$ is the mass fraction of urea in the urea aqueous solution, which is 32.5%; $M_{NH_{3}}$ is the molar mass of NH_3_, which is 17g.mol^-1^; $M_{Urea}$ is the molar mass of urea, 60g.mol^-1^.

Therefore, as in Equation 6:

$Q_{NH_{3}}=Q_{Urea}\left[ml/h\right]\times \left(1/3600\right)\left[h/s\right]\times 1.09\left[g/ml\right]\times 1000\left[mg/g\right]\times 32.5\%\times \left(2\times 17/60\right)$


$$=0.05576Q_{Urea}\left[mg/s\right]$$ (6)

The Solid SCR system’s ACU injects NH_3_ based on a formula, while the Urea SCR system’s urea solution returns to the tank via an added pipe. In Slave Control Mode, only the Solid SCR system’s reductant is injected.

### Data processing method

Work-based Window Method is not only an alternative method for in-vehicle emission testing in vehicle-mounted emission testing, but more importantly, it provides possibilities for in-service compliance inspection of heavy-duty diesel vehicles that cannot be tested by the NTE Method. No matter whether the engine is working at low speed or low load, the work-based window method can be used to evaluate the vehicle emission performance. Therefore, for the operating characteristics of urban vehicles in China, the work-based window method is used to analyze the emission characteristics of in-use vehicles, which has a wider application prospect. The discharge test result of the work-based window method is to calculate the average of the moving window of the discharge based on the reference work of the bench test cycle. The reference work of the bench test cycle is selected as the ETC cycle work or WHTC cycle work. In the calculation process, the instantaneous work of each sampling point is accumulated to reach the window work according to the sampling time series starting from the first second of the test data, and the average specific emission value of all sampling points in the work-based window is calculated. The calculated result is called the first specific emission of the work-based window. The movement interval of the power-based window is 1 second. Then, the moving window accumulates from the second of data to the specific emission of the window of power calculation, and the moving window ends at all sampling points. In this way, the average specific emission value of a series of power-based windows is obtained. The window work is determined by Equation 7 below:


$$W_{act,i}=W(t_{2,i})-W(t_{1,i})\geq W_{ref}$$ (7)

where *i* refers to the *i*th power base window; $W_{act,i}$ is the actual work of the *i*th power-based window, in kWh.window^-1^; $W(t_{1,i})$ is the cumulative work of the engine from the start time of the data to the end time t_1,_ the unit is kWh; $W_{ref}$ is the bench test transient cyclic reference work, in kWh; t_2, i_ is the end time of the *i*th window, and the end time of the window must meet the requirements of the following Equation 8:


$$W(t_{2,i}-\Delta t)-W\left(t_{1,i}\right)<W_{ref}\leq W\left(t_{2,i}\right)-W\left(t_{1,i}\right)$$ (8)

were Δt is the sampling period, equal to or less than 1 second. The mass emission of gaseous pollutants in the window is calculated according to the following formulas 9 and 10 (assuming that the density of the exhaust gas is 1.3 kg.m^-3^ under the temperature and pressure of 0.2°C and 101.3kPa, respectively):


$$m_{gas}=u_{gas}\times \sum_{j=1}^{j=n}C_{gas,j}\times q_{mew,j}\times \frac{L}{f}$$ (9)

where $m_{gas}$ is the mass emission of gaseous pollutants, the unit is g. window^-1^; $u_{gas}$ is the gaseous pollutant coefficient, which is taken as 0.001586 for NOx emissions from heavy-duty diesel engines; $C_{gas,j}$ is the concentration of gaseous pollutants in the raw exhaust gas in ppm; qmew,j is the instantaneous exhaust flow of the engine, in kg.s^-1^; *f* is the sampling frequency in Hz.

Then, the pollutant specific emission of each work-based window is calculated as following Equation 10:


$$e_{gas}=\frac{m_{gas}}{W_{act,i}}$$ (10)

where $e_{gas}$ is the power-based window ratio emission of pollutants, the unit is g. kWh^-1^.

In the on-board test, the specific pollutant emission of the Urea SCR system and the Solid SCR system was calculated by using the work-based window method. Since the WHTC cycle will be introduced into the China VI heavy-duty diesel engine emission regulations by 2018, WHTC cycle work is selected as the reference work of the power-based window in the calculation process. During the test, real-time engine speed and torque values were taken from the engine ECU to calculate the instantaneous power of the engine, and the sampling frequency was 1Hz.

## Results and discussion

### Data collection and NOX correlation analysis

Table 4 shows the test parameters that need to be measured and recorded in real-time during the on-board test. The data sources of the test parameters are also listed in the table. The emission data obtained in the test were sorted out with the engine speed and torque data, and the instantaneous data of each measurement parameter were processed according to the collected CO_2_ concentration data, to eliminate the test error caused by the different response time of the measurement data and ensure the accuracy of the data.

**Table 4:** Real-time data collected from on-board test and data sources.

Measurement parameters	Unit	Data Sources
NOx concentration ^*^	ppm	NOx sensor, GAS PEMS
CO_2_ concentration ^*^	ppm	GAS PEMS
Atmospheric temperature	°C	GAS PEMS
Atmospheric pressure	kPa	GAS PEMS
Atmospheric humidity	%	GAS PEMS
Exhaust flow	Kg.h^-1^	EFM
Exhaust gas temperature	°C	EFM
Number of particles	#	PN PEMS
Catalyst temperature	°C	Temperature Sensor
Engine speed	rpm	Engine ECU
Engine torque	Nm	Engine ECU
Engine fuel consumption	g.s^-1^	Engine ECU
Engine cooling water temperature	°C	Engine ECU
Engine intake air temperature	°C	Engine ECU
Speed	Km.h^-1^	GPS
Vehicle longitude	degree	GPS
Vehicle Latitude	degree	GPS

During the test, to evaluate the emission reduction effect of the Urea SCR system and the Solid SCR system on engine NOx emission under real-world driving conditions, the NOx sensor and GAS PEMS were used in the on-board test to measure the NOx concentration before and after the SCR catalytic converter. To ensure the consistency of the measurement values of the NOx sensor and GAS PEMS, it is necessary to conduct a correlation analysis on the NOx values of the two kinds of measuring instruments and adopt the linear regression method to perform linear fitting on the measurement of NOx sensor. For the measurement value of downstream NOx sensor, due to the cross-sensitivity of NOx sensor to NH_3_, when there is NH_3_ emission in the exhaust, the sensor reading will be inaccurate, especially when the Solid SCR system is activated for on-board test because the Solid SCR system has more effective reducing agent sprayed into the exhaust at low temperature. The injection of NH_3_ may be excessive, resulting in NH_3_ emissions, leading to inaccurate measurement values of downstream NOx sensors. Therefore, in the actual calculation process of NOx emissions, the measurement data of downstream NOx sensors are discarded, and the measurement values of upstream NOx sensors and GAS PEMS are respectively used in the calculation of NOx emissions in the upstream and downstream catalytic converter.

To verify the correlation between the NOx sensor measurements upstream of the SCR catalyst and the downstream GAS PEMS readings, a supplementary experiment was conducted to verify the correlation between the two sets of data, under the premise that the SCR system was inoperative. The analysis results show that the correlation coefficient between the two sets of data is R^2^=0.9, indicating that there is a high correlation between the engine NOx emission data measured by the upstream NOx sensor and the NOx emission measured by the downstream GAS PEMS. Perform linear regression analysis on the upstream NOx sensor measurement values according to the regression equation. The upstream NOx sensor measurements after regression analysis are used to calculate the actual engine NOx emission values, and the GAS PEMS measurements are directly used to calculate the NOx emissions after the catalyst.

### Vehicle driving characteristics

Since the type of road can significantly affect the emission performance of motor vehicles (Zhang,2022), when the test vehicle is running, its running speed changes due to the change in road traffic conditions on the test route.

As can be seen from the above figure, during the on-board test operation of the test vehicle, except that the test vehicle in the high-speed road section has a relatively simple operation condition due to the small traffic flow on the road, the vehicle operation condition of the other road sections is more complicated. To describe the driving characteristics of the test vehicle, the time distribution ratio of the idling condition, acceleration condition, deceleration condition, and cruising condition of the test vehicle in the running process and the average running speed of the vehicle are counted. The criterion for each condition is: When the speed and acceleration of the sampling point are both zero, it is the idle speed condition; when the acceleration of the sampling point is greater than or equal to 0.1ms^-2^, it is the acceleration condition; when the acceleration of the sampling point is less than or equal to -0.1ms^-2^, it is the deceleration condition. When the absolute value of acceleration is less than 0.1ms^-2^ and the speed is not zero, it is the cruising condition. Table 5 summarizes the driving characteristics of the test vehicle during the on-board test.

**Table 5:** Test vehicle driving characteristics.

Test loop	Time Proportion (%)				average speed
	idle speed	acceleration	deceleration	cruise	km.h^-1^
Urea SCR system	24.9	22.9	18.3	33.9	18.2
Solid SCR System	23.2	20.3	16.6	39.8	18.6

**Fig 1:**
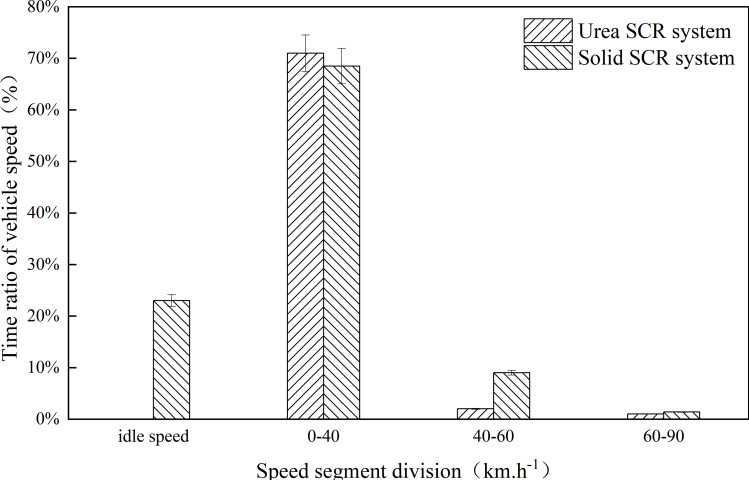
Time distribution ratio of vehicle test speed. Detailed data are shown in S1 Fig.

Judging from the time distribution ratio of the driving state of the test vehicle, the test vehicle has a similar time distribution ratio of driving characteristics in the two tests. The driving process of the vehicle includes idle speed, acceleration, and deceleration conditions with a large time proportion. The proportion of time is less than 40%. Since the test route is mainly on urban roads, the traffic flow of urban roads is large, the distribution of road intersections is dense, and the traffic conditions are complex so that the vehicles are frequently in the state of idling and acceleration and deceleration during the test process, and the time proportion of cruising conditions is low. Vehicles are often in a stop-and-go driving state, resulting in low average vehicle running speeds. The average vehicle speeds of the test vehicles in the Urea SCR system test and the Solid SCR system test were 18.2km.h^-1^ and 18.6km.h^-1^, respectively. Fig 1 analyzes the time distribution ratio of the test vehicle speed. Generally, typical urban driving speed is usually between 20–30 km.h^-1^, especially in the case of heavy traffic and dense road intersections. When the average speed of the test vehicle is 18.2 km.h^-1^ and 18.6 km.h^-1^, the average speed is lower than the typical urban driving speed. It reflects the complexity of urban traffic and frequent idle, acceleration and deceleration states.

As can be seen from (Fig 1) the time proportion of 40–60km.h^-1^ speed and 60–90km.h^-1^ speed distribution of the test vehicle is very low in the on-board test process, and the total driving time distribution proportion is less than 10%. Most of the time, the vehicle speed is distributed at idle speed and 0–40km.h^-1^ speed, in which the time distribution proportion of the Urea SCR system test and the Solid SCR system test in the idle stage is 24.9% and 23.2%, respectively. The time distribution ratios of the Urea SCR system test and the Solid SCR system test at 0–40km.h^-1^ speed ranges were 71.2% and 68.3%, respectively, resulting in lower average vehicle speeds in the two tests.

### Engine load level and catalyst distribution

**Fig 2:**
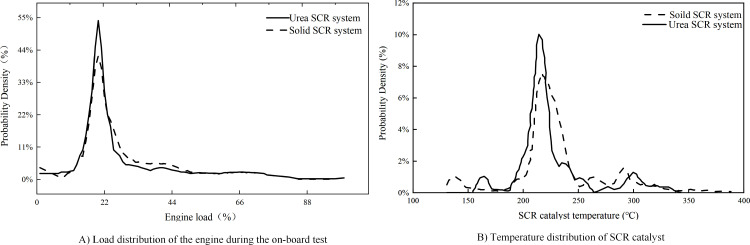
Changes in engine curves during in-vehicle testing. Detailed data are shown in S2 Fig.

As the engine load level has a significant impact on the exhaust temperature [37], and the exhaust temperature directly determines the NOx conversion performance of the SCR system, the engine load level becomes an important factor affecting the SCR performance of the SCR system. Fig 2 analyzes the load level distribution of the engine during the onboard test.

As can be seen from the figure, the engine load level of the Urea SCR system test and the Solid SCR system test in the on-board test presented a single-peak distribution. In the Urea SCR system test and the Solid SCR system test, the average load level of the engine is 24.6% and 25.3% respectively, and the load level of more than 75% of the engine operating conditions is lower than 30%, indicating that the engine runs in the low-load area most of the time in the on-board test. The low load level of the engine results in lower average exhaust temperatures. Fig 2 shows the temperature distribution characteristics of SCR catalyst in on-board test.

Upstream and downstream temperature sensors are installed on the upstream and downstream SCR catalyst to measure the exhaust temperature of the upstream and downstream SCR catalyst, and the SCR catalyst temperature is defined as the arithmetic means of the exhaust temperature measured by the upstream and downstream temperature sensor. In the on-board test, the maximum catalyst temperatures of the Urea SCR system test and the Solid SCR system test were 342 °C and 394 °C, respectively, and the average catalyst temperatures were 222.3 °C and 229.9 °C, respectively. The reasons for the average catalyst temperature in the Solid SCR system test being slightly higher than that in the Urea SCR system test are as follows: First, the hydrolysis reaction of urea is endothermic; while the evaporation and the endothermic reaction of urea aqueous solution reduce the exhaust temperature, the pyrolysis reaction further reduces the exhaust temperature. The catalyst at 200 °C–400 °C is the high conversion efficiency range of the catalyst, so it will not have much impact on NOx conversion, and the exhaust temperature of the vehicle runs under the same condition. Studies have shown (Duan,2021) that when urea aqueous solution is sprayed into the exhaust pipe, the discharge temperature will decrease by roughly 10–20 °C due to urea decomposition and endothermic absorption. Secondly, the average engine load level in the Solid SCR system test is slightly higher than that in the Urea SCR system test, which also results in the average catalyst temperature is slightly higher than that in the Urea SCR system test. According to the temperature distribution of SCR catalyst, 65.1% and 64.6% of the catalyst temperatures were in the range of 200–240 °C in the process of the Urea SCR system test and the Solid SCR system test, respectively.

### Pollutant emission results of the work-based window method

**Fig 3:**
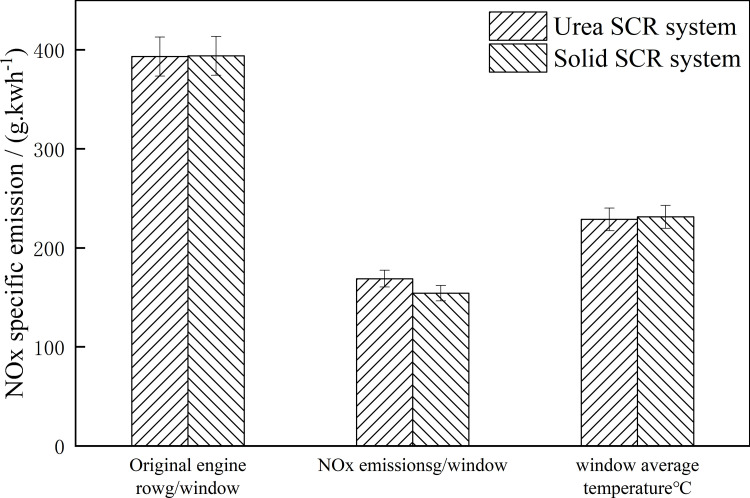
Work-based window NOx mass emission and window average catalyst temperature. Detailed data are shown in S3 Fig.

To evaluate the NOx emission control efficiency of the Urea SCR system and the Solid SCR system at low temperature, the original NOx emission, NOx emission, and window average catalyst temperature of the engine during the test were calculated by using the work-based window method. Fig 3 shows the emission test results of the vehicle test.

**Fig 4:**
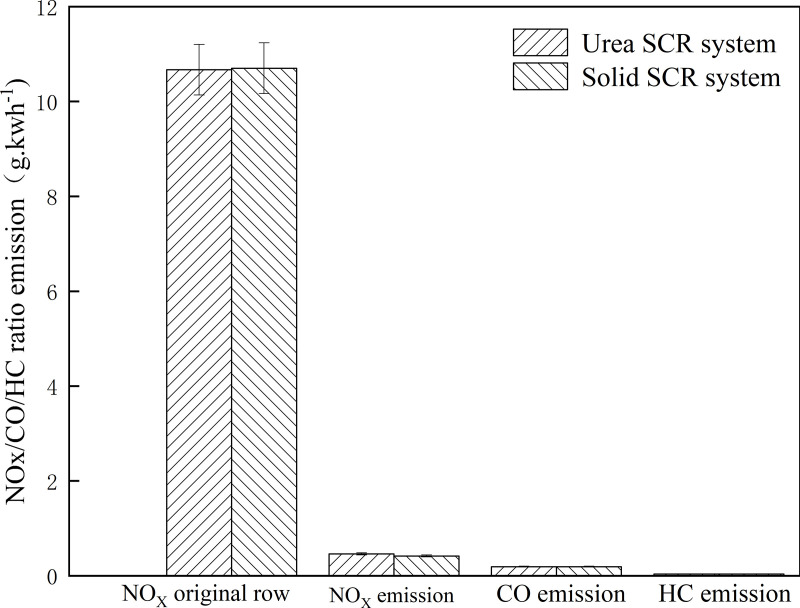
NO_x_/CO/HC ratio emission by the work-based window method. Detailed data are shown in S4 Fig.

**Fig 5:**
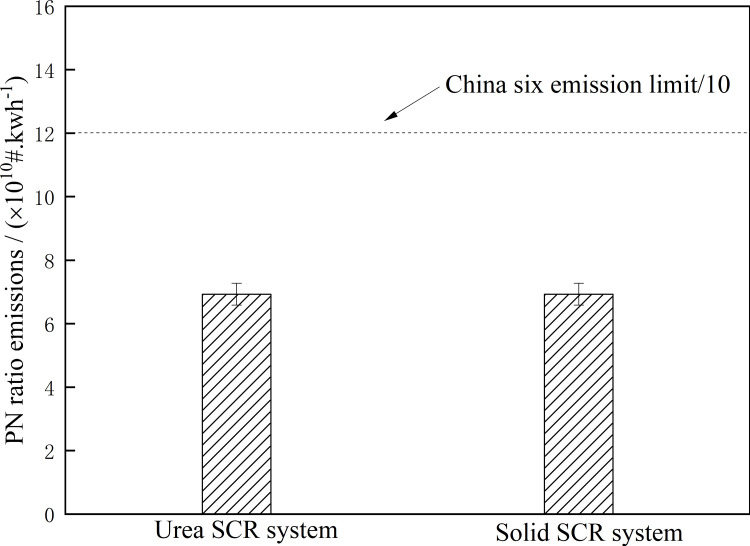
Power-base window method PN ratio emission. Detailed data are shown in S5 Fig.

As can be seen from the figure, the original NOx mass emission of the engine in the two tests was the same. The average window mass emission of NOx in the engine’s original row in the Urea SCR system test and the Solid SCR system test were 393.2g. Window ^-1^ and 393.9 g.window^-1^, respectively. The mean window NOx emission of the Urea SCR system is higher than that of the Solid SCR system, 168.9 g.window^-1,^ and 154.2 g.window^-1^, respectively. The average window temperatures of the two tests were 228.8°C and 231.3°C, respectively. This is because the average temperature of the catalyst in the test is low. Under the condition of low temperature, since urea cannot be completely decomposed into reducing agent NH_3_, the amount of effective reducing agent in the Urea SCR system is less than that in the Solid SCR system under the same ammonia nitrogen ratio injection. As a result, the engine’s original row is the same. The NOx mass emission of the Urea SCR system is higher than that of the Solid SCR system. The experimental results show that the NOx emission control performance of the Urea SCR system is lower than that of the Solid SCR system at low temperatures. NOx/CO/HC specific emission in the power base window is shown in Fig.4:

As can be seen from Fig 4, the average value of NOx power-based window ratio of the engine in the Urea SCR system test and the Solid SCR system test is 10. 67g.kWh^-1^ and 10.7g.kWh^-1^ respectively. Under real-world driving conditions, the NOx emission values of the Urea SCR system and the Solid SCR system were 0.5and 0.4g.kWh^-1^, while CO and HC emissions were 0.2 and 0.04 g. kWh^-1^, respectively. The average NOx conversion efficiency of the Urea SCR system is 95.6%, and that of the Solid SCR system is 96.1%. The average NOx conversion efficiency of the Solid SCR system is 0.5% higher than that of the Urea SCR system under the operating condition of low exhaust temperature. The NOx emission decreased from 0.5 g.kWh^-1^ to 0.4 g. kWh^-1^ in the Urea SCR system and the NOx emission decreased by 8.9%. The experimental results show that, compared with the Urea SCR system, the NOx conversion efficiency of the Solid SCR system is increased by 0.5% and NOx emission is reduced by 8.9% under low-temperature driving conditions. Fig.5 shows the PN ratio emission in the power-based window.

It can be seen from Fig 5 that the PN power-based window ratio of the Urea SCR system and the Solid SCR system is the same, that is, whether the Urea SCR system or the Solid SCR system is used, its influence on PN value is not significant, and both are lower than the PN limit required by the China VI emission regulations.

#### Study on pollutant emission factors of real-worlds.

**Fig 6:**
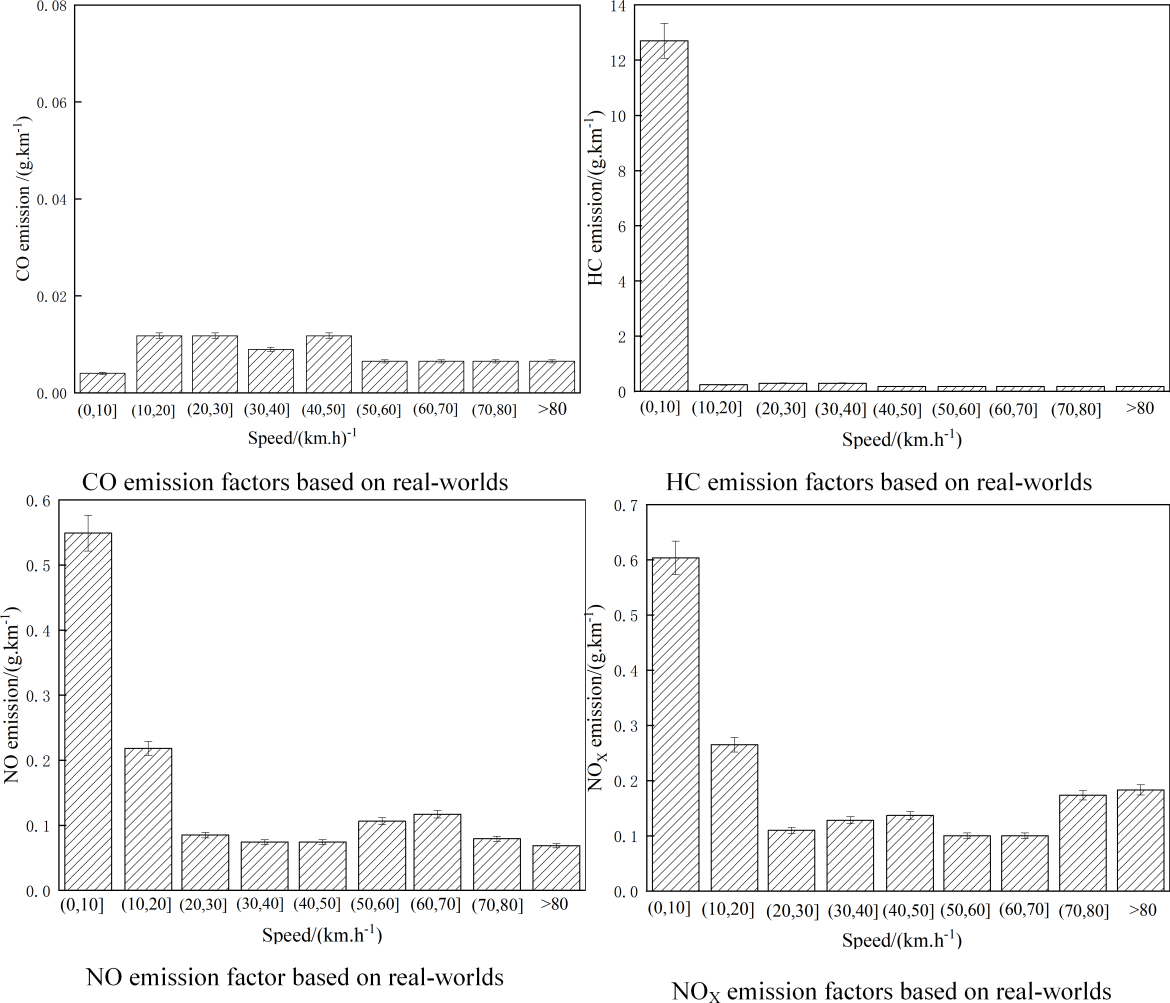
Emission factors based on real-worlds. Detailed data are shown in S6 Fig.

Based on the real-world pollutant emission factors of the Solid SCR, the equal-interval separation method based on vehicle speed is adopted. The method is to divide the real-world speed of tested vehicles into 9 different equal-speed intervals, and study pollutant emission factors such as NO_X_, CO, and HC according to different equal-speed intervals. The results are shown in Fig.6.

As can be seen from Fig 6, in the speed range (0,10), CO, HC, NO, NO_X,_ and other pollutant emission factors are in the highest state. As the engine has just started, the exhaust temperature is low, and the after-treatment device has not fully played its role. With the increase in vehicle speed, pollutant emission factors gradually decrease and tend to be relatively stable. Among the pollutant emission factors studied, HC, NO, and NO_X_ pollutant emission factors decrease greatly with the increase in vehicle speed, while CO emission factors decrease relatively little. The reasons are as follows: DOC is installed in the heavy-duty vehicle reprocessing system that meets the emission standards of China VI, which can control CO at a low level at low speeds; At the beginning of the test, the NO_X_ emission factor was at a high level because the air-fuel ratio and combustion temperature at this time created good conditions for its generation. After that, due to the increase in exhaust temperature, the SCR system came into play and transformed NO_X_, reducing the NO_X_ emission factor. When the vehicle starts to accelerate, more fuel is injected into the cylinder and the pollutant conversion efficiency is not high, resulting in a large amount of HC generation. When the vehicle speed exceeds 20km. h^-1^, HC pollutant emission is reduced or even close to zero.

#### Advantages of the Solid SCR technology.

**Fig 7:**
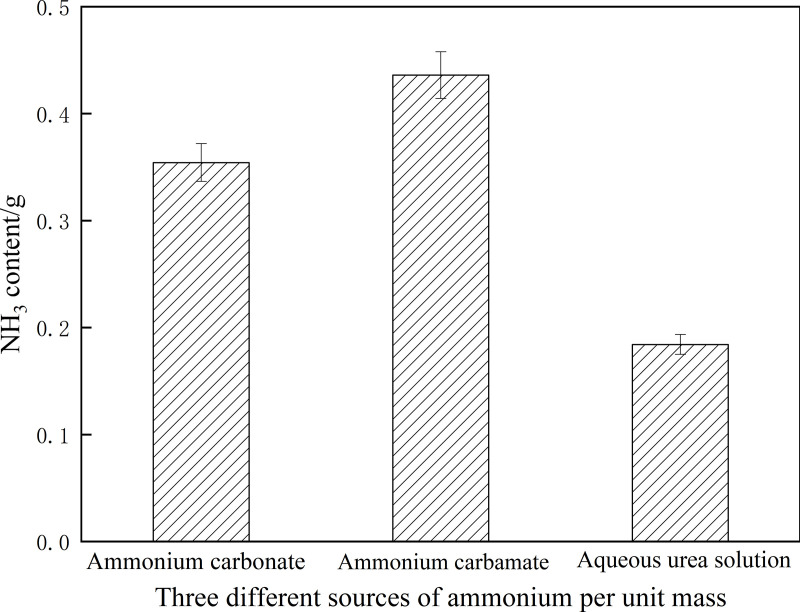
Ammonia produced by different ammonium sources per unit mass. Detailed data are shown in S7 Fig.

For the unit mass of ammonium carbonate, ammonium carbamate, and urea aqueous solution, ammonium carbamate and ammonium carbonate produce more NH_3_ than the urea aqueous solution. The NH_3_ produced by the unit mass of different ammonium salts is shown in Fig.7.

According to the above characteristics, NH_3_ can be stored in a closed container in the form of solid ammonium salt in advance, and the temperature of the closed container can be controlled by the system control unit to release NH_3_ and seal it in the closed container, and then quantitative injection of NH_3_ into the exhaust and exhaust according to the requirements of the operating conditions of the diesel engine, to achieve the purpose of controlling NOx emissions. From the above analysis, it can be concluded that the Solid SCR technology has the following characteristics:

The reducing agent NH_3_ is sprayed into the exhaust pipe. The Solid SCR technology directly releases NH_3_ into the exhaust and reacts with NOx. The production process of NH_3_ does not depend on engine exhaust temperature and can work at low exhaust temperature conditions where the Urea SCR technology cannot work, which is conducive to improving the low-temperature emission performance of diesel vehicles and reducing carbon emissions of diesel vehicles.

The reducing agent sprayed is gaseous NH_3_, so no crystallization will occur in the exhaust pipe path. The main reason for the deposition of the Urea SCR system in the exhaust pipe is that the urea solution injected into the exhaust pipe generates biuret and cyanic acid after the complex reaction between the products of pyrolysis and hydrolysis reaction and urea under the high-temperature environment of exhaust. Since the Solid SCR technology is directly injected into the exhaust pipe with pure gaseous NH_3_, the application of the Solid SCR technology can completely solve the problem of deposition produced by the Urea SCR technology at low-temperatures.

The reducing agent NH_3_ is stored in a solid state and will not be frozen at low-temperatures. A Solid SCR system does not have the defect of freezing at low-temperatures, which is beneficial to be used in cold regions of China.

Ammonia storage density is high. Under the same storage volume, the Solid SCR system can carry more effective reducing agent NH_3_, so the mileage after refueling is higher than that of the Urea SCR system. Because the Solid SCR system reduces the volume of the after-treatment system and can effectively reduce the ignition temperature of the SCR system catalyst, it can be used in light diesel vehicles in the city.

To sum up, the Solid SCR technology can solve the defects of current Urea SCR technology such as low-temperature activity deficiency, exhaust pipe path crystallization, low-temperature icing, and so on. The volume density of ammonia carried by the Solid SCR technology is like that of pure liquid ammonia, and it can carry a more effective reducing agent than the Urea SCR system under the same volume. It is a promising NOx emission control technology for diesel engines.

### Related research comparison

**Fig 8:**
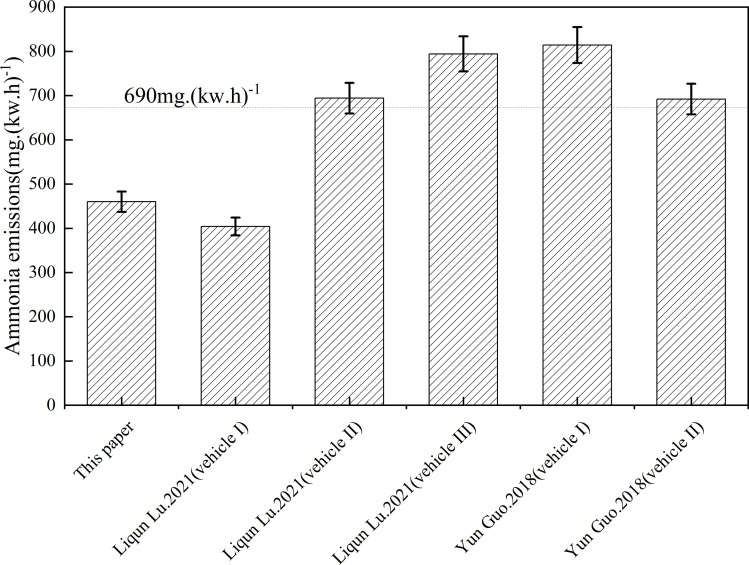
Comparison of relevant literature data. Detailed data are shown in S8 Fig.

To obtain the real and reliable experimental data, this paper compares and analyzes the real-world emission and fuel economy of Lv Liqun’s China VI heavy diesel vehicle based on the power-based window method [38] and Guo Yong’s bus based on different fuels [39]. The specific parameters of test vehicle I, test vehicle II and test vehicle III selected by Lv Liqun are shown in Table 6, and the main parameters of test vehicle I and test vehicle II selected by Guo Yong are shown in Table 7. The comparison of experimental data in relevant literature is shown in Fig. 8.

**Table 6:** Experimental vehicle parameters.

Number	Displacement (L)	Power (kW)	Aftertreatment technology	Quality of equipment (kg)
Vehicle I	5.1	170	DOC+DPF+SCR	>12000
Vehicle II	12.6	410	DOC+DPF+SCR	>12000
Vehicle III	12.4	353	DOC+DPF+SCR	>12000

**Table 7:** Experimental vehicle parameters.

Number	Displacement(L)	Power (kW)	Aftertreatment technology	Quality of equipment (kg)
Vehicle I	8.4	243	DOC+DPF+SCR	18000
Vehicle II	8.4	243	DOC+DPF+SCR	18000

It is obvious from the above Fig 8 that the average ammonia emission data obtained in this experiment and the average ammonia emission data obtained by Lv Liqun Test Vehicle I are lower than the limit value of the China VI emission standard, while the average ammonia emission data obtained by Lv Liqun Test Vehicle II, Test Vehicle III, Guo Yong Test Vehicle I and Test Vehicle II are higher than the limit value of the China VI emission standard. The analysis results show that, on the one hand, because of the large proportion of urban roads section in the test section of Lvliqun, the traffic flow is large, the test vehicle is in the state of walking and stopping, and the idle speed ratio is high, the engine load is low, the temperature is lower than the hydrolysis temperature of urea, the SCR system can not play a normal role, so that the ammonia emission increases and the average ammonia emission is higher than the China VI limit; on the other hand, the test diesel used by Guo Yong contains high sulfur content, which reduces the catalyst effect in some SCR systems and leads to high average ammonia emission, exceeding the China VI limit.

## Conclusion

On-board test a China VI diesel truck, the emission Aftertreatment SCR system using the Urea SCR technology and the Solid SCR technology respectively studied the pollutant emission of urban vehicles under the real-world driving conditions under the same injection strategy. The test results show that:

1) Due to the large traffic flow in real-world conditions, dense distribution of road intersections, and complex traffic conditions, the test vehicles are frequently in idle and acceleration and deceleration state during the driving process, and the running time of vehicles in idle stage and 0–40km.h^-1^ speed is more than 90%, resulting in low average running speed of vehicles. The average vehicle speed was 18. 2km.h^-1^ and 18. 6km.h^-1^, respectively.
2) During the test, the average load level of the engine was relatively low. The average load level of the engine in the Urea SCR system test and the Solid SCR system test was 24.6% and 25.3%, respectively. The load of the engine in 75% operating conditions was lower than 30%, leading to the engine running in the low load region in most of the test conditions.
3) In the actual on-board road test, the average temperature of the SCR catalyst was 222.3°C and 229.9°C for the Urea SCR system test and the Solid SCR system test, respectively, because of the average speed of the vehicle was low and the engine often ran under low speed and low load condition.
4) The results of the work-based window method show that: Under the real-world operation conditions, the NOx conversion efficiency of the Urea SCR system and the Solid SCR system with the same injection strategy is increased by 0.5% and the NOx emission is reduced by 8.9% compared with the Urea SCR system when the window average temperature is 228.8°C and 231.3°C. For PN emission, no matter the Urea SCR system or the Solid SCR system is selected, it has little influence on PN emission.
5) In the study of real-world pollutant emission factors based on a Solid SCR system, CO, HC, NO, NO_X,_ and other pollutant emission factors gradually decrease and tend to be relatively stable with the increase in vehicle speed.
6) The Solid SCR technology can resolve the Urea SCR technology activity insufficiency, the exhaust line crystallization at low temperature, low-temperature freezing, the Solid SCR technology with ammonia volume density and the pure liquid ammonia, under the same volume, can carry a more effective reducing agent than the Urea SCR system, is a very promising diesel engine NOx emission control technologies.
7) Compared with the research data in relevant literature, the experimental data of ammonia emission obtained in this paper are lower than the emission limit of China VI, and the data are true and reliable.
8) Solid SCR technology is able to effectively reduce NOx emissions at low temperatures, making it suitable for urban light-duty diesel vehicles, which is important for more stringent emission standards in the future (e.g. China’s VI emission standard). Moreover, due to the higher ammonia storage density of the Solid SCR system, it means that it can carry more effective reductant NH3 in the same volume, which not only prolongs the refuelling mileage, but also reduces the volume of the aftertreatment system and improves the overall future performance of the vehicle, which has a wide range of impacts, especially in the application in urban low-speed driving conditions.

## Supporting information

S1 Fig Time distribution ratio of vehicle test speed. S1 Table is the S1 Fig legend.
(PDF)

S2 Fig Changes in engine curves during in-vehicle testing. S2 Table is the S2 Fig legend.
(PDF)

S3 Fig Work-based window NOx mass emission and window average catalyst temperature. S3 Table is the S3 Fig legend.
(PDF)

S4 Fig NOx/CO/HC ratio emission by the work-based window method. S4 Table is the S4 Fig legend.
(PDF)

S5 Fig Power-base window method PN ratio emission. S5 Table is the S5 Fig legend.
(PDF)

S6 Fig Emission factors based on real-worlds. S6 Table is the S6 Fig legend.
(PDF)

S7 Fig Ammonia produced by different ammonium sources per unit mass. S7 Table is the S7 Fig legend.
(PDF)

S8 Fig Comparison of relevant literature data. S8 Table.docx is the S8 Fig legend.
(PDF)
