# Supplementary material for: Research on real-world emission characteristics based on the Symmetry Solid SCR system
Source: PLoS One. 2025 Apr 29;20(4):e0320323. doi: 10.1371/journal.pone.0320323 (PMC12040118; doi:10.1371/journal.pone.0320323)
Supplement: S1 Fig — S1 Table is the S1 Fig legend. (PDF) [file pone.0320323.s001.pdf]

S1 Table1 Time distribution ratio of vehicle test speed

Speed band division km/h	Vehicle Speed Distribution % of Time		Vehicle Speed Distribution % of Time	
	Urea SCR System	5% of Speed Division Time	Percentage Solid SCR System	5% of Speed Division Time Percentage
idle speed			23	1.15
0-40	71	3.55	68.5	3.425
40-60	2	0.1	9	0.45
60-90	1	0.05	1.4	0.07
