# Supplementary material for: Research on real-world emission characteristics based on the Symmetry Solid SCR system
Source: PLoS One. 2025 Apr 29;20(4):e0320323. doi: 10.1371/journal.pone.0320323 (PMC12040118; doi:10.1371/journal.pone.0320323)
Supplement: S2 Fig — S2 Table is the S2 Fig legend. (PDF) [file pone.0320323.s002.pdf]

S2 Table Changes in engine curves during in-vehicle testing

	SCR catalyst temperature (℃)		Engine load（%）	
Probability Density（%）	3.14074	0.05789	4.56506	0.07072
	10.58602	0.05986	10.58602	0.04736
	19.06716	0.0671	15.89483	0.0546
	23.98752	0.10821	27.15986	0.11019
	26.83615	0.15821	36.02945	0.39307
	30.3322	0.22466	34.92885	0.39307
	30.00849	0.27137	31.04435	0.27663
	32.46867	0.3536	39.20179	0.2786
	32.46867	0.3286	43.08628	0.19078
	32.85712	0.42728	51.89113	0.10986
