# Supplementary material for: Research on real-world emission characteristics based on the Symmetry Solid SCR system
Source: PLoS One. 2025 Apr 29;20(4):e0320323. doi: 10.1371/journal.pone.0320323 (PMC12040118; doi:10.1371/journal.pone.0320323)
Supplement: S3 Fig — S3 Table is the S3 Fig legend. (PDF) [file pone.0320323.s003.pdf]

S3 Table Work-based window NOx mass emission and window average catalyst temperature

NOx specific emission / (g.kwh-1)				
Original engine rowg/window	393.24	19.662	393.9	19.695
NOx emissionsg/window	168.85	8.4425	154.2	7.71
window average temperature℃	228.8	11.44	231.3	11.565
