# Supplementary material for: Research on real-world emission characteristics based on the Symmetry Solid SCR system
Source: PLoS One. 2025 Apr 29;20(4):e0320323. doi: 10.1371/journal.pone.0320323 (PMC12040118; doi:10.1371/journal.pone.0320323)
Supplement: S4 Fig — S4 Table is the S4 Fig legend. (PDF) [file pone.0320323.s004.pdf]

S4Table NOx/CO/HC ratio emission by the work-based window method

NOx, CO, HC emissions / (g/kwh)				
NOx Original Emission	10.67	10.7	0.5335	0.535
NOx Emission	0.461	0.419	0.02305	0.02095
CO emission	0.192	0.192	0.0096	0.0096
HC emission	0.038	0.038	0.0019	0.0019
