# Supplementary material for: Research on real-world emission characteristics based on the Symmetry Solid SCR system
Source: PLoS One. 2025 Apr 29;20(4):e0320323. doi: 10.1371/journal.pone.0320323 (PMC12040118; doi:10.1371/journal.pone.0320323)
Supplement: S5 Fig — S5 Table is the S5 Fig legend. (PDF) [file pone.0320323.s005.pdf]

**S5 TAble** Power-base window method PN ratio emission

| PN specific emissions / (g/kwh) | | |
|---|---|---|
| Urea SCR Systems | 6.925816549 | 0.346290827 |
| Solid state SCR system | 6.925816549 | 0.346290827 |
