# Supplementary material for: Research on real-world emission characteristics based on the Symmetry Solid SCR system
Source: PLoS One. 2025 Apr 29;20(4):e0320323. doi: 10.1371/journal.pone.0320323 (PMC12040118; doi:10.1371/journal.pone.0320323)
Supplement: S6 Fig — S6 Table is the S6 Fig legend. (PDF) [file pone.0320323.s006.pdf]

S6 table Emission factors based on real-worlds

	Speed/(kw.h)-1			
CO emission/(g.km^{-1})	0.46	23	36.02	0.39
HC emission/(g.km^{-1})	0.40	20.2	34.92	0.39
NO emission/(g.km^{-1})	0.69	34.7	30.00	0.27
NOx emission/(g.km^{-1})	0.79	39.7	32.47	0.35
