# Supplementary material for: Research on real-world emission characteristics based on the Symmetry Solid SCR system
Source: PLoS One. 2025 Apr 29;20(4):e0320323. doi: 10.1371/journal.pone.0320323 (PMC12040118; doi:10.1371/journal.pone.0320323)
Supplement: S7 Fig — S7 Table is the S7 Fig legend. (PDF) [file pone.0320323.s007.pdf]

S7 Table Ammonia produced by different ammonium sources per unit mass

	NH3 content/g	
Ammonium carbonate	0.3542	0.01771
Ammonium Carbamate	0.4359	0.021795
Urea aqueous solution	0.1842	0.00921
