# Supplementary material for: Research on real-world emission characteristics based on the Symmetry Solid SCR system
Source: PLoS One. 2025 Apr 29;20(4):e0320323. doi: 10.1371/journal.pone.0320323 (PMC12040118; doi:10.1371/journal.pone.0320323)
Supplement: S8 Fig — S8 Table.docx is the S8 Fig legend. (PDF) [file pone.0320323.s008.pdf]

S8 Table Comparison of relevant literature data

	Ammonia emissions(mg.(kw.h)-1)	
This paper	460	23
Liqun Lu.2021(vehicle I)	404	20.2
Liqun Lu.2021(vehicle II)	694	34.7
Liqun Lu.2021(vehicle III)	794	39.7
Yun Guo.2018(vehicle I)	814	40.7
Yun Guo.2018(vehicle II)	692	34.6
